# *FMR1* Allelic Complexity and IVF Fertilization Success: Limitations and Future Perspectives

**DOI:** 10.3390/ijms26125752

**Published:** 2025-06-16

**Authors:** Bárbara Rodrigues, Emídio Vale-Fernandes, Vanessa Sousa, Isabel Marques, Rosário Santos, António J. A. Nogueira, Paula Jorge

**Affiliations:** 1 Molecular Genetics Laboratory, Laboratory Genetics Service, Genetics and Pathology Clinic, Unidade Local de Saúde de Santo António (ULSSA), Largo Professor Abel Salazar, 4099-001 Porto, Portugal; be_rodrigues_@hotmail.com (B.R.); vanessa_sousa4d@hotmail.com (V.S.); isabel.marques@chporto.min-saude.pt (I.M.); rosario.santos.cgm@chporto.min-saude.pt (R.S.); 2 UMIB—Unit for Multidisciplinary Research in Biomedicine, ICBAS—School of Medicine and Biomedical Sciences, UPorto—University of Porto, Rua Jorge de Viterbo Ferreira 228, 4050-313 Porto, Portugal; emidiovalefernandes.pma@chporto.min-saude.pt; 3 ITR—Laboratory for Integrative and Translational Research in Population Health, Rua das Taipas 135, 4050-600 Porto, Portugal; 4 Centre for Medically Assisted Procreation/Public Gamete Bank, Gynaecology Departament, Centro Materno-Infantil do Norte Dr. Albino Aroso (CMIN), Unidade Local de Saúde de Santo António (ULSSA), Largo da Maternidade Júlio Dinis 45, 4050-651 Porto, Portugal; 5 CESAM—Centre for Environmental and Marine Studies, Department of Biology, University of Aveiro, Campus Universitario de Santiago, 3810-193 Aveiro, Portugal; antonio.nogueira@ua.pt

**Keywords:** *FMR1* allelic complexity, *FMR1* gene, predictor, oocytes with two pronuclei, fertilization success, ovarian reserve, assisted reproductive technology

## Abstract

We investigated whether *FMR1* allelic complexity—integrating CGG repeat length with the number and pattern of AGG interspersions—can be used as a predictor of ovarian reserve and in vitro fertilization (IVF) success. This cohort study included 124 females with infertility attributed to female factors undergoing intracytoplasmic sperm injection (ICSI). The total CGG repeat lengths and AGG interspersion patterns of the *FMR1* gene were determined by conventional polymerase chain reaction (PCR) and triplet-primed PCR. The allelic complexity (*allelic score*) was calculated using a previously described formula by combining the *allelic scores*, allowing for the stratification of samples into *equivalent* and *dissimilar* groups. No statistically significant differences were observed in ovarian reserve markers or overall IVF outcomes between the two groups. However, within the *dissimilar* group, the *allelic score* of allele 1 was significantly correlated with the number of both injected metaphase II and two-pronuclei oocytes. These findings suggest that *FMR1* allelic complexity may contribute to predicting IVF success, particularly in females classified in the *dissimilar* group, who appear more susceptible to IVF failure than those in the *equivalent* group. Further research into the predictive utility of *FMR1* could provide valuable insights for fertility assessment and enhance assisted reproductive technologies.

## 1. Introduction

The fragile X messenger ribonucleoprotein 1 (*FMR1*) gene, located on the long arm of the X chromosome at Xq27.3, plays a critical role in female reproductive health [1]. The *FMR1* gene is notably associated with female infertility through its relationship with fragile X-associated primary ovarian insufficiency (FXPOI; OMIM #311360) [2,3]. Approximately 20% of female carriers of the *FMR1* premutation (PM, 55 < CGG repeats < 200) develop FXPOI [4,5]. These carriers typically exhibit hypergonadotropic hypogonadism and present with absent or irregular menstrual cycles before age 40, thereby increasing their risk of infertility [6,7,8].

The *FMR1* gene contains a polymorphic region within its 5′ untranslated region (UTR). Based on the number of CGG repeats, alleles are classified as normal (CGG repeats < 45), intermediate (45–54 CGG repeats), PM (55–199 CGG repeats), and full mutation (≥200 CGG repeats) [9]. Intermediate alleles were associated with idiopathic primary ovarian insufficiency (POI) [10,11]. On the other hand, some studies have implicated normal-sized alleles—particularly those with fewer than 26 CGG repeats—in reduced in vitro fertilization (IVF) pregnancy rates, poor embryonic quality, diminished ovarian response to stimulation, decreased ovarian reserve (DOR), and POI [12,13,14,15,16], although other research has failed to confirm these associations [17,18,19,20]. Most studies into *FMR1*’s role in DOR have primarily considered the total CGG repeat length. Considering that most non-expanded alleles (CGG repeats < 55) are typically interrupted by stabilizing AGG triplets, which commonly occur at positions 9 or 10 within the CGG stretches, we previously developed a formula that determines the allelic complexity—*allelic score*—of each *FMR1* allele. This score integrates both the total CGG repeat length and the number and pattern of these AGG interruptions [21]. It is well established that both the length of the CGG repeat tract and the pattern of AGG interruptions influence repeat stability and the risk of expansion in offspring. Specifically, AGG interruptions are known to act as stabilizing elements, mitigating strand slippage during DNA replication. Consequently, alleles lacking these protective AGG interruptions exhibit a significantly higher propensity for expansion, particularly during maternal transmission [22,23]. While the influence of AGG interruptions on clinical outcomes in individuals with a premutation remains an area of active investigation, with conflicting findings reported regarding their role in FXPOI [4,24], we hypothesize that a comprehensive metric—one that considers the entire repeat region, encompassing both its length and the AGG interspersion pattern—may provide a more accurate assessment of risk and contribute to a clearer understanding of these complex relationships.

In this study, we aimed to evaluate whether *FMR1* allelic complexity could serve as a predictor of ovarian reserve and IVF success. To this end, we analyzed samples from females experiencing infertility who were undergoing intracytoplasmic sperm injection (ICSI). Samples were categorized based on their *FMR1* allelic complexity, and correlations with ovarian reserve markers and IVF outcomes were assessed.

## 2. Results

### 2.1. Demographic and Clinical Characteristics of the Study Cohort

This study included 124 females diagnosed with infertility, with a mean age of 34.7 ± 3.7 years, ranging from 22 to 40 years. Most females presented multiple etiologies of infertility, the main causes being ovulatory dysfunction (*n* = 83, 66.8%), endometriosis (*n* = 17, 13.7%), and oocyte factor (*n* = 12, 9.7%). Less frequently (*n* = 12, 9.7%), they presented hypothyroidism, hyperprolactinemia, POI, DOR, and adenomyosis. Table 1 shows the demographic and clinical characteristics of the study cohort.

### 2.2. FMR1 Gene Repeat Region Characterization

**Figure 1:**
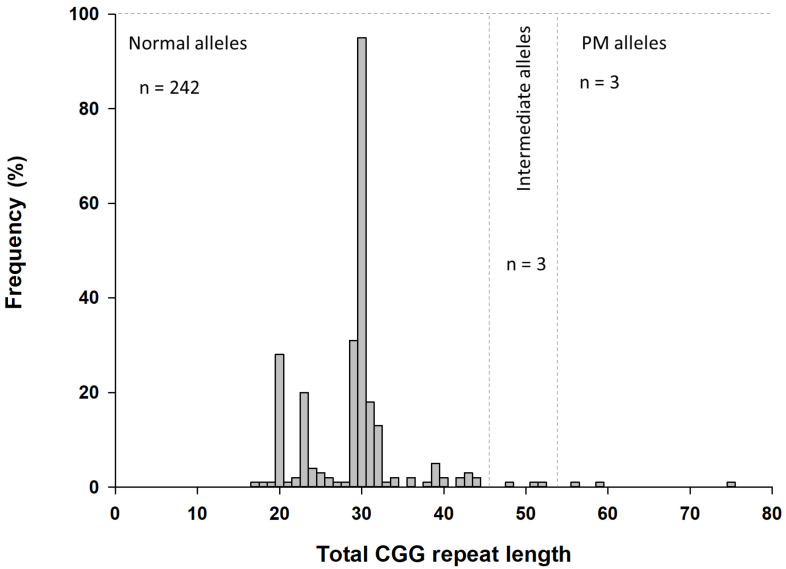
Distribution of the total CGG repeat length in the study cohort. The most frequent alleles presented 30 CGG repeats, followed by 29 and 20 CGG repeats. *n*—number of alleles; PM—premutation.

Of the 124 samples analyzed, 118 presented two normal-sized alleles, ranging from 17 to 44 CGG repeats. Three samples exhibited an intermediate allele with 48, 51, and 52 CGG repeats, while three other samples presented a PM allele with 56, 59, and 75 CGG repeats, respectively (Figure 1).

Across the total of 248 alleles, the most frequent CGG repeat length was 30 CGG repeats (*n* = 95, 38.3%). Most alleles presented one or two AGG interruptions (*n* = 232, 93.6%), while 4% of alleles (*n* = 10) presented no AGG interruptions, and 2.4% of alleles (*n* = 6) presented three AGG interruptions. Overall, 62 distinct AGG interspersion patterns were identified, with the most common being (CGG)_10_AGG(CGG)_9_AGG(CGG)_9_ (*n* = 81, 32.7%) and (CGG)_9_GG(CGG)_9_AGG(CGG)_9_ (*n* = 25, 10.1%). *FMR1* molecular data can be found in Supplementary Table S1.

The PM alleles were excluded from further analysis, as the study focused on normal-sized and intermediate alleles.

### 2.3. FMR1 Allelic Scores Combination and Comparison of Mathematical Models

The summary of the *FMR1* allelic complexity (*allelic score*) results can be found in Table 2. The mean *allelic score* was 125.5 ± 95.6 for allele 1 and 198.9 ± 135.4 for allele 2.

The combination of *allelic scores* resulted in two distinct groups: one containing alleles with similar *allelic scores* (*equivalent* group) and another containing alleles with *different allelic scores* (*dissimilar* group). These were classified as follows: when both alleles presented an *allelic score* > 150 or < 150, the sample was included in the *equivalent* group; when one allele presented an *allelic score* > 150 and the other < 150, the sample was included in the *dissimilar* group. The correlation between the *allelic scores* of each group was described using a linearized logarithmic model (or mathematical model). Significant correlations were found in the *equivalent* group: r = 0.562; df = 67; *p* < 0.0001, and in the *dissimilar* group: r = −0.417; df = 50; *p* = 0.0021. *Allelic scores* above 700 were obtained in samples with 3 AGG interruptions (*n* = 3 in both the *equivalent* and *dissimilar* groups, Supplementary Table S2, samples 4, 14, 44, 71, 89, and 97, respectively), due to the relevance attributed to the number of AGG interruptions by the formula.

ANCOVA was then used to compare the regression models resulting from the combination of the *allelic scores* of both alleles in each group. Coincident regression lines demonstrated no statistically significant differences in *equivalent* (F _(2, 139)_ = 0.3023; *p* = 0.7396) and *dissimilar* (F _(2, 99)_ = 0.3496; *p* = 0.7058) groups when comparing this infertile cohort with the reference set of potentially fertile females (described in Rodrigues et al. (2020) [21]). These results enable the development of a more robust mathematical model that includes all observations: *equivalent* group—Score 2 = −334.6 + 106.6 × ln(score 1) (r = 0.547; df = 141; *p* < 0.0001) and *dissimilar* group—Score 2 = 482.5 − 73.6 × ln(score 1) (r = −0.874; df = 101; *p* < 0.0001).

### 2.4. Markers of Ovarian Reserve and IVF Outcomes According to Stratification of FMR1 Allelic Complexities

**Figure 2:**
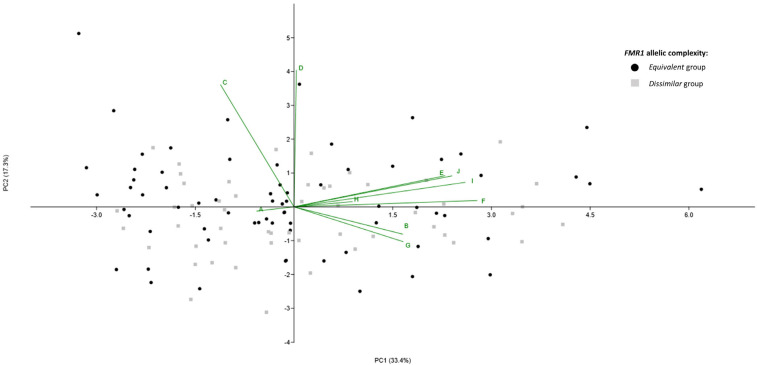
Multivariate statistical analysis results of data from *FMR1 equivalent* and *dissimilar* groups. The black circles represent samples from the *equivalent* group, while the grey squares represent samples from the *dissimilar* group. The green lines represent vectors corresponding to the original variables, indicating their contribution and direction in the PCA space. The markers of ovarian reserve considered were FSH (A), AMH (B), and total dose of gonadotrophins (C). The IVF outcomes considered were stimulation days (D), number of follicles on the trigger day (E), number of retrieved oocytes (F), number of immature oocytes (G), number of aberrant oocytes (H), number of injected MII oocytes (I) and number of oocytes with 2PN (J).

PCA was conducted to evaluate if the markers of ovarian reserve and IVF outcomes were able to discriminate between the *equivalent* and *dissimilar* groups. The variables analyzed did not allow a distinct separation between the two groups, as shown in Figure 2. The first principal component (PC1) accounted for 33.4% of the total variance, while the second principal component (PC2) explained an additional 17.3% of the variance. Among the variables analyzed, the number of retrieved oocytes, the number of injected MII oocytes, and the number of 2PN oocytes were identified as the primary contributors to the variance explained by PC1.

Consistent with these findings, no statistically significant differences were found between the *equivalent* and *dissimilar* groups in any of the markers of ovarian reserve and IVF outcomes analyzed (*p* > 0.05 for all the variables) (Table 3).

### 2.5. Association of FMR1 Allelic Complexity with Markers of Ovarian Reserve and with IVF Outcomes

We next explored if the *FMR1 allelic score* of each individual allele correlated with the markers of ovarian reserve and with the IVF outcomes. In the *equivalent* group, no significant correlations were observed between the *allelic score* of allele 1 or allele 2 and the markers of ovarian reserve and IVF outcomes (*p* > 0.05) (Table 4 and Supplementary Table S3). Similarly, no significant correlations were found for allele 2 in the *dissimilar* group (Supplementary Table S3). However, in this group, a significant negative correlation was observed between the *allelic score* of allele 1 and the number of injected MII oocytes (Pearson correlation: r = −0.289, *n* = 49, *p* = 0.044) as well as the number of 2PN oocytes (Pearson correlation: r = −0.311, *n* = 48, *p* = 0.031) (Table 4). Additionally, a significant positive correlation was found between the number of injected MII oocytes and the number of oocytes with 2PN (Pearson correlation: r = 0.859, *n* = 48, *p* < 0.001).

**Figure 3:**
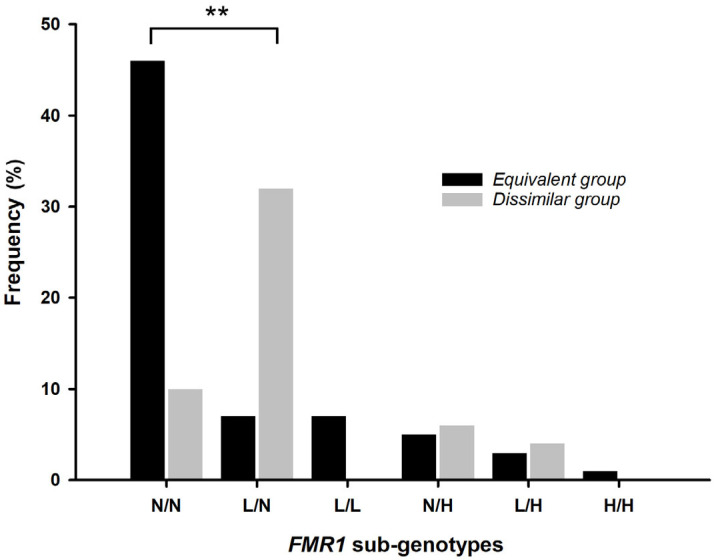
*FMR1* sub-genotype distributions in the *equivalent* and *dissimilar* groups. The black bars represent the samples of the *equivalent* group, while the grey bars represent the samples of the *dissimilar* group. Sub-genotypes: N/N—Normal/Normal; L/N—Low/Normal; L/L—Low/Low; N/H—Normal/High; H/H—High/High; L/H—Low/High; ** *p* < 0.001.

The most frequent CGG repeat length of allele 1 of the *dissimilar* group was 20 CGG repeats (Supplementary Table S2). Considering that alleles with fewer than 26 CGG repeats have been previously associated with poor fertility prognosis, we analyzed the distribution pattern of the sub-genotypes previously described [25] among the two groups (Figure 3). Most samples from the *dissimilar* group presented alleles with < 26 CGG repeats (*n* = 36, 69.2%), while most samples from the *equivalent* group presented alleles with > 26 CGG repeats (*n* = 52, 75.4%). A statistically significant difference was found in the distribution of the normal/normal and low/normal sub-genotype (χ^2^ = 35.9; df = 1; *p* < 0.001).

## 3. Discussion

In this study, we aimed to investigate whether the *FMR1* gene allelic complexity can be used as a predictor of ovarian reserve and IVF success. By combining *FMR1 allelic scores*, we introduce a novel integrative approach that extends beyond traditional CGG repeat length assessment, offering a more comprehensive evaluation of the repetitive tract. This approach allowed us to categorize samples according to allelic complexity and explore their relationship with ovarian reserve markers and IVF outcomes.

The mathematical model derived from the combination of the *allelic scores* of our infertile cohort and was not statistically different from the reference models calculated in the previous study using samples from potentially fertile females [21]. The fact that the combination of the allelic complexity is independent of the clinical condition in each group of samples allows the integration of data from both studies, permitting the development of a more robust model.

Notably, within the *dissimilar* group (where the allelic complexity of each allele is inversely related), the *allelic score* of allele 1 showed a negative correlation with the number of 2PN oocytes, a key indicator of successful fertilization [26,27,28]. Specifically, cases with an *allelic score* of allele 1 exceeding 150 exhibited very few 2PN oocytes, suggesting a higher risk of fertilization failure in this subgroup. Conversely, a female with a low *allelic score* in allele 1 (*allelic score* = 23; Supplementary Table S2, sample 114) demonstrated high fertilization success (12 out of 13 injected MII oocytes resulting in2PN; Supplementary Table S4, sample 114). In contrast, another female with a high *allelic score* (*allelic score* = 205; Supplementary Table S2, sample 112) had poor fertilization outcomes, with only 3 out of 7 injected MII oocytes reaching the 2PN stage, alongside three unfertilized and one degenerated oocyte (Supplementary Table S4, sample 112). Interestingly, the size range of 35–54 CGG repeats has been previously associated with an increased risk of DOR [29,30], which aligns with our observation that 5 out of 7 samples with high *allelic score* (>150) in allele 1 also presented with a number of CGG repeats above 34 in allele 2. These findings suggest that patterns of allelic complexity—beyond mere CGG repeat counts—may serve as novel predictive biomarkers for fertilization failure risk in IVF, representing a significant advancement from previous studies that focused solely on CGG repeat length. This approach provides insights that may clarify the inconsistent associations previously reported between CGG repeat numbers and IVF outcomes.

Our observation that the categorization of samples based on *FMR1* allelic complexity, using our established formula, might elucidate the disparities seen in COC and 2PN oocyte numbers in PCOS versus non-PCOS populations [31] presents a new perspective on interpreting existing data. While elevated AMH levels in the *dissimilar* group, echoing findings in PCOS [31], did not reach statistical significance in our cohort, the trend suggests a potential link that warrants further investigation within the framework of allelic complexity. Consistent with prior studies linking shorter CGG repeats (<26) to poorer IVF outcomes [12,13,14,16,32,33], our *dissimilar* group was enriched with such alleles. However, our key innovation lies in demonstrating that allelic complexity allows for the identification of a specific subgroup at elevated risk of fertilization failure, even within these shorter repeat ranges. This underscores the critical importance of considering not just the number of CGG repeats but also the stabilizing pattern of AGG interruptions. This is further supported by Quilichini et al. (2024), finding that allelic complexity enhances POI risk prediction in intermediate and premutated alleles [34], extending the significance of this metric beyond fertilization outcomes to broader reproductive health. The discrepancy with Nunes et al. (2024) study, which found no association between CGG repeat number and IVF outcomes [35], directly highlights the added value of our allelic complexity assessment. By incorporating the AGG interruption pattern, our more comprehensive approach appears to capture nuances in the *FMR1* alleles that are missed by simply considering the total CGG repeat length.

Additionally, a recent publication by our team highlights the importance of molecular profiling, such as hormone and metabolite markers, in elucidating the mechanisms underlying PCOS-related reproductive dysfunctions. It demonstrates that integrating molecular data can identify biomarkers linked to mitochondrial and glycolytic impairments, which may adversely affect oocyte quality and fertilization success [36]. This supports our approach of combining genetic complexity with phenotypic indicators, thereby strengthening the case for the potential predictive value of *FMR1* allelic complexity in IVF outcomes.

In summary, our study’s primary novel contribution is the identification of *FMR1* allelic complexity patterns, particularly within the *dissimilar* group, as a promising predictive biomarker for fertilization success in IVF. This refined approach offers a more nuanced understanding compared to traditional CGG repeat analysis and may help to identify females at higher risk of fertilization failure. Furthermore, our findings reinforce the broader clinical utility of assessing *FMR1* allelic complexity, as suggested by others [34].

While our primary focus was on the relationship between *FMR1* allelic complexity and fertilization outcomes, our analysis of the study population also revealed an important secondary finding: an increased frequency of PM carriers (3 out of 124 cases) compared to the general population [37,38]. This finding underscores the critical need for the implementation of routine FXS screening protocols in infertile females, aligning with recommendations from other researchers [39,40,41], to facilitate informed reproductive decisions and genetic counseling.

While acknowledging the limitations of our small sample size and exclusive focus on fertilization outcomes, our findings strongly advocate for future research with larger cohorts to validate the predictive power of *FMR1* allelic complexity for the entire spectrum of IVF success, including embryo development and live birth rates. Ultimately, a deeper understanding of *FMR1* allelic complexity holds significant potential for developing more personalized and effective IVF treatments.

## 4. Materials and Methods

### 4.1. Study Design and Participants

This was a cohort study that included 124 females with infertility attributed to female factors and who were undergoing ICSI. Females with tubal obstruction were excluded from the study. Females were recruited from the Centre for Medically Assisted Procreation, Centro Materno-Infantil do Norte Dr. Albino Aroso (CMIN), Unidade Local de Saúde de Santo António (ULSSA) between January 2020 and February 2022.

This study was conducted in accordance with the Declaration of Helsinki and was approved by the Ethics Committee of the ULSSA (process number 2020.119(097-DEFI/099-CE). All participants provided written informed consent.

### 4.2. Demographic and Clinical Data

Data regarding the age and cause of infertility was obtained for all participants. Data on markers of ovarian reserve, including follicle-stimulating hormone (FSH) at day 3, anti-Müllerian hormone (AMH), and antral follicle count (AFC), were obtained. Data regarding IVF outcomes, including response to ovarian stimulation (total gonadotrophin dose, stimulation duration, follicle count on trigger day, and the number of retrieved, immature and aberrant oocytes), oocyte maturation (number of injected metaphase II [MII] oocytes), and fertilization success (number of two pronuclei [2PN] oocytes) were also obtained from all participants. All data were obtained from clinical records.

### 4.3. FMR1 CGG Repeat Region Analysis

#### 4.3.1. Total CGG Repeat Length

The number of CGG repeats was determined using a fluorescent polymerase chain reaction (PCR). The forward primer P1-5′-TTCGGTTTCACTTCCGGTG-3′ and the reverse primer P2- FAM labeled—5′-CCATCTTCTCTTCAGCCCTGC-3′ were used. All the PCR components used are detailed in Supplementary Table S5. The amplification protocol involved an initial incubation at 98 °C for 5 min, followed by 42 cycles of denaturation at 95 °C for 1 min and 10 s, annealing at 57 °C for 45 s, and extension at 68 °C for 1 min and 10 s, with a final extension of 10 min at 68 °C. PCR products were analyzed by capillary electrophoresis using ABI PRISM^®^ 3130xl Genetic Analyzer (Applied Biosystems™, Foster City, CA, USA) with 500 ROX™ size standard (GeneScan™, Warrington, UK) and were further analyzed using GeneMapper^®^ software version 4.0 (Applied Biosystems™) (Supplementary Figure S1A). A previously sequenced sample containing 30 CGGs was used as a control.

The categorization of *FMR1* alleles followed the European Molecular Genetics Quality Network (EMQN) guidelines: normal (CGG repeats < 45), intermediate or “grey-zone” (45 ≤ CGG repeats ≤ 54), PM (55 ≤ CGG repeats < 200), and full mutation (CGG repeats ≥ 200) [9].

#### 4.3.2. AGG Interspersion Pattern

AGG interruptions were determined by Triplet-Primed PCR (TP-PCR) using the forward primer P1-5′-GACGGAGGCGCCGCTGCCAGG-3′, reserve primer P2- HEX labeled—5′-TACGCATCCCAGTTTGAGACGGGCCGCCGCCGC-3′, and primer “tail” P3- 5′-ACGCATCCCAGTTTGAGACG-3′. All the PCR components are described in Supplementary Table S6. Amplification involved an initial incubation at 98 °C for 5 min, followed by 15 cycles of denaturation at 98 °C for 1 min, annealing at 55 °C for 1 min, and extension 68 °C for 2 min, and 30 cycles of denaturation at 98 °C for 1 min, annealing at 55 °C for 1 min, and extension 68 °C for 3 min. The final extension of 10 min was performed at 68 °C. PCR products were analyzed using capillary electrophoresis as previously described. The AGG interspersion pattern was analyzed using the software described above. In samples where the AGG interspersion pattern was ambiguous (*n* = 34 samples), the FRAXA PCR kit LabGscan™ (Diagnostica Longwood, Zaragoza, Spain) was used, following the manufacturer’s specifications (Supplementary Figure S1B).

#### 4.3.3. *FMR1* Allelic Complexity

The allelic complexity for each allele was calculated using the formula described by Rodrigues et al. (2020) [21], which integrates both the number and pattern of AGG interspersions and the total CGG repeat length. The resulting value, termed the *allelic score*, quantitatively reflects the complexity of the *FMR1* CGG/AGG substructure.

For instance, consider an allele with the AGG interspersion pattern CGG_10_AGGCGG_9_AGGCGG_9._ To calculate its *allelic score*, the formula considers the length of each uninterrupted CGG repeat stretch and its position relative to the AGG interruptions. The *allelic score* calculation emphasizes the 5′ end of the allele by assigning higher weights to CGG repeats closer to the 5′ end and decreasing weights to repeats located further downstream towards the 3′ end from the initial AGG interruptions. Following Rodrigues et al. (2020) [21], the *allelic score* for this specific pattern is calculated as follows:

*Allelic score* = (Number of CGG repeats before the 1st AGG × 4^1 − 1^) +  (Number of CGG repeats between the 1st and 2nd AGG × 4^2 − 1^) +  (Number of CGG repeats after the last AGG × 4^2^)

*Allelic score* = (9 × 4^1 − 1^) + (9 × 4^2 − 1^) + (10 × 4^2^) = 205

#### 4.3.4. *FMR1* Sub-Genotypes

The categorization of samples according to *FMR1* sub-genotypes was based on the study by Gleicher et al. (2010a) as follows [42]: normal/normal (N/N) when both alleles were within the new normal range (between 26 and 34 CGG repeats); low/normal (L/N), when one allele was below and the other within the normal range; normal/high (N/H), when one allele was within the normal range and the other was above; low/high (L/H), when one allele was below and the other was above the normal range; low/low (L/L), when both alleles were below the normal range; and high/high (H/H), when both alleles were above the normal range.

### 4.4. Statistical Analysis

Categorical variables are presented as absolute (n) and relative frequencies (%). Continuous variables are presented as the mean ± standard deviation (SD) and range, as indicated in each table. A linearized form of a logarithmic model [i.e., regression of ln(score 1) against score 2] was used to obtain a mathematical model to predict the allelic complexity (*allelic score*) relationships between both alleles for each group. By analysis of covariance (ANCOVA), we compared the regression models between potentially fertile females (i.e., the reference set that was previously described by Rodrigues et al. (2020) [21] and infertile females, following the methodology outlined by Zar [43]. Principal component analysis (PCA) was performed to test if the markers of ovarian reserve and IVF outcomes were able to discriminate between *equivalent* and *dissimilar* groups. For normally distributed data, a comparative analysis of the markers of ovarian reserve and IVF outcomes between the *equivalent* and *dissimilar* groups was performed using a *t*-test. When the data failed normality and homoscedasticity tests, comparisons were performed using the Mann-Whitney test. Pearson correlations were used to assess the relationship between the *allelic score* of each allele, the markers of ovarian reserve and IVF outcomes. The chi-square test was used to compare the number of samples (frequency) across the sub-genotype categories between the two groups (*equivalent* and *dissimilar*).

All statistical analyses were performed with SigmaPlot version 14.0 (Systat Software^®^ Inc., Chicago, IL, USA), except for PCA, which was performed with Past^®^ version 4.16c (Statistic software) [44]. A statistical significance level of 0.05 was used for all statistical tests.

## Figures and Tables

**Table 1:** Demographic and clinical characteristics of the study cohort (*n* = 124 unless stated otherwise).

	Study Cohort	
Characteristics	Mean ± SD	Range
Age (years)	34.7 ± 3.7	22–40
Markers of ovarian reserve		
Day 3 FSH (mUI/mL)	8.4 ± 11.8 (*n* = 118)	3.3–40.0
AMH (ng/mL)	3.1 ± 2.9 (*n* = 123)	0.1–18.9
AFC	7.4 ± 3.5 (*n* = 76)	1.0–16.0
IVF outcomes		
Response to ovarian stimulation		
Total dose of gonadotrophins (IU/mL)	26,582.0 ± 885.3 (*n* = 122)	1025.0–7200.0
Stimulation duration (days)	10.2 ± 1.9 (*n* = 123)	5.0–16.0
Number of follicles on the trigger day	7.2 ± 4.8 (0–21.0) (*n* = 122)	0–21.0
Number of retrieved oocytes	11.7 ± 8.5 (*n* = 122)	0–46.0
Number of immature oocytes	2.0 ± 2.2 (*n* = 120)	0–10.0
Number of aberrant oocytes	0.8 ± 1.6 (*n* = 124)	0–12.0
Oocyte maturation		
Number of injected MII oocytes	8.1 ± 5.7 (*n* = 120)	0–28.0
Fertilization success		
Number of 2PN oocytes	5.3 ± 4.0 (*n* = 116)	0–17.0

2PN, two pronuclei; AFC, antral follicle count; AMH, anti-Müllerian hormone, FSH, follicle-stimulating hormone; MII, metaphase II; *n*, number of samples; SD, standard deviation.

**Table 2:** Summary of the *FMR1* allelic complexity (*allelic score*) of the study cohort.

	Allele 1 (Shorter CGG Repeat Length)	Allele 2 (Longer CGG Repeat Length)
Number of alleles	121	121
*Allelic score*		
Mean ± SD	125.5 ± 95.6	198.9 ± 135.4
Median (range)	61.0 (23–765)	205.0 (23–829)
Most frequent (*n*, %)	205 (34, 28.1)	205 (46, 38.0)
	49 (26, 21.5)	206 (11, 9.1)
	189 (16, 13.2)	201 (10, 8.3)

**Table 3:** Comparison of the markers of ovarian reserve and IVF outcomes between *equivalent* and *dissimilar* groups.

	*Equivalent* Group (*n* = 69 ^a^)	*Dissimilar* Group (*n* = 52 ^a^)	*p*-Value ^b^
**Age (years)**	34.7 ± 3.9	34.6 ± 3.3	0.550
Markers of ovarian reserve			
Day 3 FSH (mUI/mL)	7.3 ± 2.3 (*n* = 65)	9.8 ± 17.7 (*n* = 51)	0.676
AMH (ng/mL)	2.9 ± 2.4 (*n* = 64)	3.6 ± 3.4 (*n* = 46)	0.238
AFC ^c^	6.9 ± 3.3 (*n* = 40)	7.9 ± 3.3 (*n* = 33)	0.205
IVF outcomes			
Response to ovarian stimulation			
Total dose of gonadotrophins (IU/mL)	2811.8 ± 1032.9 (*n* = 68)	2452.5 ± 620.5 (*n* = 51)	0.075
Stimulation duration (days)	10.4 ± 2.0 (*n* = 68)	9.9 ± 1.8	0.08 ^d^
Number of follicles on the trigger day	6.9 ± 4.7 (*n* = 68)	7.6 ± 5.0 (*n* = 51)	0.451
Number of retrieved oocytes	11.4 ± 9.0 (*n* = 68)	12.0 ± 7.6 (*n* = 51)	0.411
Number of immature oocytes	1.9 ± 2.0 (*n* = 68)	2.4 ± 2.2 (*n* = 49)	0.694
Number of aberrant oocytes	0.9 ± 1.8 (*n* = 68)	0.7 ± 1.5 (*n* = 49)	0.334
Oocyte maturation			
Number of injected MII oocytes	8.0 ± 6.1 (*n* = 68)	8.2 ± 5.0 (*n* = 49)	0.578
Fertilization success			
Number of 2PN oocytes	5.4 ± 4.0 (*n* = 64)	5.1 ± 3.9 (*n* = 48)	0.690

2PN, two pronuclei; AFC, antral follicle count; AMH, anti-Müllerian hormone; FSH, follicle-stimulating hormone; MII, metaphase II; *n*, number of samples; SD, standard deviation; ^a^ Samples size used unless stated otherwise. Values are presented as mean ± SD; ^b^ *p*-value of statistical test (Mann-Whitney Test unless stated otherwise); ^c^ Variables excluded from PCA; ^d^ *p*-value of the *t*-Test.

**Table 4:** Correlation between the *allelic score* of allele 1, makers of ovarian reserve, and IVF outcomes for *equivalent* and *dissimilar* groups.

	*Equivalent* Group			*Dissimilar* Group		
Clinical Characteristics	Pearson’s Correlation Coefficient	*p*-Value	*n*	Pearson’s Correlation Coefficient	*p*-Value	*n*
Markers of ovarian reserve						
Day 3 FSH (mUI/mL)	−0.068	0.588	65	−0.053	0.713	51
AMH (ng/mL)	−0.029	0.819	64	−0.082	0.589	46
AFC	0.015	0.925	40	−0.045	0.806	33
IVF outcomes						
Response to ovarian stimulation						
Total dose of gonadotrophins (IU/mL)	0.096	0.437	68	0.135	0.343	51
Stimulation duration (days)	0.010	0.937	68	−0.078	0.581	52
Number of follicles on the trigger day	0.237	0.0512	68	−0.050	0.726	51
Number of retrieved oocytes	0.060	0.627	68	−0.153	0.283	51
Number of immature oocytes	0.139	0.257	67	−0.168	0.249	49
Number of aberrant oocytes	−0.019	0.876	68	−0.094	0.523	49
Oocyte maturation						
Number of injected MII oocytes	0.0794	0.520	68	−0.289	**0.044**	49
Fertilization success						
Number of 2PN oocytes	0.044	0.731	64	−0.311	**0.031**	48

2PN, two pronuclei; AFC, antral follicle count; AMH, anti-Müllerian hormone; FSH, follicle-stimulating hormone; MII, metaphase II; *n*, number of samples; Statistically significant results are marked in bold.

## Data Availability

All data are contained within the article or Supplementary Material.

## Supplementary Materials

The following supporting information can be downloaded at: https://www.mdpi.com/article/10.3390/ijms26125752/s1.

## Abbreviations

AFC	Antral follicle count
AGG	Adenine-Guanine-Guanine
AMH	Anti-Müllerian hormone
ANCOVA	Analysis of covariance
CGG	Cytosine-Guanine-Guanine
DOR	Diminished ovarian reserve
*FMR1*	Fragile X messenger ribonucleoprotein 1
FSH	Follicle-stimulating hormone
FXPOI	Fragile X-associated primary ovarian insufficiency
IVF	In vitro fertilization
ICSI	Intracytoplasmic sperm injection
MII	Metaphase II
PC1	First principal component
PC2	Second principal component
PCR	Polymerase chain reaction
PM	Premutation
POI	Primary ovarian insufficiency
PCA	Principal component analysis
SD	Standard deviation
UTR	Untranslated region
2PN	Two pronuclei
