# Supplementary material for: FMR1 Allelic Complexity and IVF Fertilization Success: Limitations and Future Perspectives
Source: Int J Mol Sci. 2025 Jun 16;26(12):5752. doi: 10.3390/ijms26125752 (PMC12193248; doi:10.3390/ijms26125752)
Supplement: Supplementary file 1 [file ijms-26-05752-s001.zip › ijms-3600827-supplementary.pdf]

Supplementary Tables

Supplementary Table S1. Summary of the *FMR1* CGG repetitive region data.

	Allele 1 (shorter CGG repeat length)	Allele 2 (longer CGG repeat length)	Both alleles
Number of alleles	124	124	248
Total CGG repeat length			
Mean ± SD	26.0 ± 4.4	32.6 ± 7.2	29.3 ± 6.8
Median (range)	29.0 (17 - 39)	30.0 (20 - 75)	30 (17 - 75)
Most frequent (*n*, %)	30 (44, 35.5)	30 (51, 41.1)	30 (95, 38.3)
	20 (26, 21.0)	31 (17, 13.7)	29 (31, 12.5)
	29 (20, 16.1)	32 (12, 10.1)	20 (28, 11.3)
Most common $(CGG)_x$ AGG patterns (*n*, %)	$(CGG)_{10}AGG(CGG)_9AGG(CGG)_9$ (35, 28.2)	$(CGG)_{10}AGG(CGG)_9AGG(CGG)_9$ (46, 37.1)	$(CGG)_{10}AGG(CGG)_9AGG(CGG)_9$ (81, 32.7)
	$(CGG)_{10}AGG(CGG)_9$ (22, 17.7)	$(CGG)_{10}AGG(CGG)_9AGG(CGG)_{10}$ (11, 8.9)	$(CGG)_9AGG(CGG)_{12}AGG(CGG)_9$ (25, 10.1)
	$(CGG)_9AGG(CGG)_9AGG(CGG)_9$ (17, 13.7)	$(CGG)_9AGG(CGG)_{12}AGG(CGG)_9$ (9, 7.3)	$(CGG)_{10}AGG(CGG)_9$ (24, 9.7)

AGG, adenine-guanine-guanine; CGG, cytosine-guanine-guanine; n, number of alleles; SD, Standard deviation; x, number of CGGs.

Supplementary Table S2. *FMR1* CGG repeat detailed data.

Sample	Allele 1 (shorter CGG repeat length)			Allele 2 (longer CGG repeat length)			*FMR1* sub-genotypes
	(CGG)$_x$AGG Pattern	Repeat length	*Allelic score*	(CGG)$_x$AGG Pattern	Repeat length	*Allelic score*	
1	(CGG)$_{23}$	23	23	(CGG)$_{12}$AGG(CGG)$_{10}$	23	58	Low/Low
2	(CGG)$_{10}$AGG(CGG)$_{9}$AGG(CGG)$_{9}$	30	205	(CGG)$_{10}$AGG(CGG)$_{9}$AGG(CGG)$_{9}$	30	205	Normal/Normal
3	(CGG)$_{9}$AGG(CGG)$_{9}$AGG(CGG)$_{9}$	29	189	(CGG)$_{10}$AGG(CGG)$_{9}$AGG(CGG)$_{9}$	30	205	Normal/Normal
4	(CGG)$_{9}$AGG(CGG)9AGG(CGG)$_{9}$AGG(CGG)$_{9}$	39	765[§]	(CGG)$_{9}$AGG(CGG)$_{9}$AGG(CGG)$_{22}$	42	202	High/High
5	(CGG)$_{10}$AGG(CGG)$_{9}$AGG(CGG)$_{9}$	30	205	(CGG)$_{10}$AGG(CGG)$_{9}$AGG(CGG)$_{9}$	30	205	Normal/Normal
6	(CGG)$_{10}$AGG(CGG)$_{9}$AGG(CGG)$_{9}$	30	205	(CGG)$_{10}$AGG(CGG)$_{9}$AGG(CGG)$_{9}$	30	205	Normal/Normal
7	(CGG)$_{10}$AGG(CGG)$_{11}$	22	51	(CGG)$_{13}$AGG(CGG)$_{16}$	30	68	Low/Normal
8	(CGG)$_{10}$AGG(CGG)$_{9}$	20	49	(CGG)$_{12}$AGG(CGG)$_{10}$	23	58	Low/Low
9	(CGG)$_{9}$AGG(CGG)$_{9}$AGG(CGG)$_{10}$	30	190	(CGG)$_{10}$AGG(CGG)$_{9}$AGG(CGG)$_{9}$	30	205	Normal/Normal
10	(CGG)$_{10}$AGG(CGG)$_{9}$	20	49	(CGG)$_{10}$AGG(CGG)$_{9}$	20	49	Low/Low
11	(CGG)$_{10}$AGG(CGG)$_{9}$AGG(CGG)$_{9}$	30	205	(CGG)$_{10}$AGG(CGG)$_{9}$AGG(CGG)$_{24}$	44	220	Normal/High
12	(CGG)$_{9}$AGG(CGG)$_{9}$AGG(CGG)$_{9}$	29	189	(CGG)$_{10}$AGG(CGG)$_{9}$AGG(CGG)$_{9}$	30	205	Normal/Normal
13	(CGG)$_{9}$AGG(CGG)$_{9}$AGG(CGG)$_{8}$	28	188	(CGG)$_{9}$AGG(CGG)$_{9}$AGG(CGG)$_{10}$	30	190	Normal/Normal
14	(CGG)$_{10}$AGG(CGG)$_{9}$AGG(CGG)$_{9}$	30	205	(CGG)$_{10}$AGG(CGG)$_{9}$AGG(CGG)$_{9}$AGG(CGG)$_{9}$	39	829[§]	Normal/High
15	(CGG)$_{9}$AGG(CGG)$_{9}$AGG(CGG)$_{9}$	29	189	(CGG)$_{9}$AGG(CGG)$_{9}$AGG(CGG)$_{24}$	44	204	Normal/High
16	(CGG)$_{9}$AGG(CGG)$_{9}$AGG(CGG)$_{9}$	29	189	(CGG)$_{10}$AGG(CGG)$_{9}$AGG(CGG)$_{9}$	30	205	Normal/Normal
17	(CGG)$_{12}$AGG(CGG)$_{10}$	23	58	(CGG)$_{34}$	34	34	Low/Normal
18	(CGG)$_{9}$AGG(CGG)$_{9}$AGG(CGG)$_{10}$	30	190	(CGG)$_{10}$AGG(CGG)$_{9}$AGG(CGG)$_{22}$	43	218	Normal/High
19	(CGG)$_{10}$AGG(CGG)$_{9}$AGG(CGG)$_{9}$	30	205	(CGG)$_{10}$AGG(CGG)$_{9}$AGG(CGG)$_{9}$	30	205	Normal/Normal
20	(CGG)$_{10}$AGG(CGG)$_{9}$AGG(CGG)$_{9}$	30	205	(CGG)$_{10}$AGG(CGG)$_{9}$AGG(CGG)$_{9}$	30	205	Normal/Normal
21	(CGG)$_{10}$AGG(CGG)$_{9}$	20	49	(CGG)$_{10}$AGG(CGG)$_{9}$	20	49	Low/Low

22	$(CGG)_{10}AGG(CGG)_9AGG(CGG)_9$	30	205	$(CGG)_{10}AGG(CGG)_9AGG(CGG)_9$	30	205	Normal/Normal
23	$(CGG)_{10}AGG(CGG)_7AGG(CGG)_{10}$	29	198	$(CGG)_{10}AGG(CGG)_9AGG(CGG)_9$	30	205	Normal/Normal
24	$(CGG)_{13}AGG(CGG)_9$	23	61	$(CGG)_{20}AGG(CGG)_9$	30	89	Low/Normal
25	$(CGG)_{10}AGG(CGG)_9AGG(CGG)_9$	30	205	$(CGG)_{10}AGG(CGG)_9AGG(CGG)_9$	30	205	Normal/Normal
26	$(CGG)_{10}AGG(CGG)_9AGG(CGG)_9$	30	205	$(CGG)_{10}AGG(CGG)_9AGG(CGG)_{10}$	31	206	Normal/Normal
27	$(CGG)_9AGG(CGG)_9AGG(CGG)_9$	29	189	$(CGG)_{10}AGG(CGG)_9AGG(CGG)_9$	30	205	Normal/Normal
28	$(CGG)_9AGG(CGG)_9AGG(CGG)_9$	29	189	$(CGG)_9AGG(CGG)_9AGG(CGG)_9$	29	189	Normal/Normal
29	$(CGG)_{10}AGG(CGG)_9$	20	49	$(CGG)_9AGG(CGG)_{21}$	31	57	Low/Normal
30	$(CGG)_{10}AGG(CGG)_9AGG(CGG)_9$	30	205	$(CGG)_9AGG(CGG)_{12}AGG(CGG)_9$	32	201	Normal/Normal
31	$(CGG)_{10}AGG(CGG)_9AGG(CGG)_9$	30	205	$(CGG)_{10}AGG(CGG)_9AGG(CGG)_{10}$	31	206	Normal/Normal
32	$(CGG)_{10}AGG(CGG)_9AGG(CGG)_9$	30	205	$(CGG)_{10}AGG(CGG)_9AGG(CGG)_{10}$	31	206	Normal/Normal
33	$(CGG)_{10}AGG(CGG)_9$	20	49	(CGG)25AGG(CGG)10	36	110	Low/High
34	$(CGG)_{10}AGG(CGG)_9AGG(CGG)_9$	30	205	$(CGG)_{10}AGG(CGG)_9AGG(CGG)_9$	30	205	Normal/Normal
35	$(CGG)_9AGG(CGG)_9AGG(CGG)_9$	30	189	$(CGG)_9AGG(CGG)_9AGG(CGG)_9$	30	189	Normal/Normal
36	$(CGG)_9AGG(CGG)_9AGG(CGG)_9$	29	189	$(CGG)_{10}AGG(CGG)_9AGG(CGG)_{10}$	31	206	Normal/Normal
37	$(CGG)_9AGG(CGG)_{10}$	20	46	$(CGG)_{10}AGG(CGG)_8AGG(CGG)_9$	29	57	Low/Normal
38	$(CGG)_9AGG(CGG)_{13}$	23	49	$(CGG)_{10}AGG(CGG)_{27}$	38	67	Low/High
39	$(CGG)_{10}AGG(CGG)_9AGG(CGG)_9$	30	205	$(CGG)_{10}AGG(CGG)_9AGG(CGG)_9$	30	205	Normal/Normal
40	$(CGG)_9AGG(CGG)_9AGG(CGG)_9$	29	189	$(CGG)_{10}AGG(CGG)_{12}AGG(CGG)_9$	33	217	Normal/Normal
41	$(CGG)_{10}AGG(CGG)_9AGG(CGG)_9$	30	205	$(CGG)_{10}AGG(CGG)_9AGG(CGG)_9$	30	205	Normal/Normal
42	$(CGG)_9AGG(CGG)_9AGG(CGG)_9$	29	189	$(CGG)_9AGG(CGG)_{12}AGG(CGG)_9$	32	201	Normal/Normal
43	$(CGG)_{10}AGG(CGG)_9AGG(CGG)_9$	30	205	$(CGG)_{10}AGG(CGG)_9AGG(CGG)_9$	30	205	Normal/Normal
44	$(CGG)_{10}AGG(CGG)_9AGG(CGG)_9$	30	205	$(CGG)9AGG(CGG)_9AGG(CGG)_9AGG(CGG)_9$	39	765§	Normal/High
45	$(CGG)_9AGG(CGG)_9AGG(CGG)_9$	29	189	$(CGG)_{10}AGG(CGG)_9AGG(CGG)_9$	30	205	Normal/Normal
46	$(CGG)_9AGG(CGG)_9AGG(CGG)_9$	29	189	$(CGG)_{10}AGG(CGG)_9AGG(CGG)_9$	30	205	Normal/Normal

47	$(CGG)_{9}AGG(CGG)_{9}AGG(CGG)_{9}$	29	189	$(CGG)_{10}AGG(CGG)_{9}AGG(CGG)_{9}$	30	205	Normal/Normal
48	$(CGG)_{10}AGG(CGG)_{9}AGG(CGG)_{9}$	30	205	$(CGG)_{9}AGG(CGG)_{12}AGG(CGG)_{9}$	32	201	Normal/Normal
49	(CGG)10AGG(CGG)9AGG(CGG)9	30	205	$(CGG)_{10}AGG(CGG)_{9}AGG(CGG)_{9}$	30	205	Normal/Normal
50	$(CGG)_{10}AGG(CGG)_{9}AGG(CGG)_{9}$	30	205	$(CGG)_{9}AGG(CGG)_{12}AGG(CGG)_{9}$	32	201	Normal/Normal
51	$(CGG)_{10}AGG(CGG)_{9}$	20	49	$(CGG)_{24}AGG(CGG)_{9}$	34	105	Low/Normal
52	$(CGG)_{9}AGG(CGG)_{9}AGG(CGG)_{9}$	29	189	$(CGG)_{10}AGG(CGG)_{9}AGG(CGG)_{9}$	30	205	Normal/Normal
53	$(CGG)_{9}AGG(CGG)_{9}AGG(CGG)_{9}$	30	189	$(CGG)_{9}AGG(CGG)_{11}AGG(CGG)_{9}$	32	197	Normal/Normal
54	$(CGG)_{9}AGG(CGG)_{9}AGG(CGG)_{10}$	30	190	$(CGG)_{9}AGG(CGG)_{10}AGG(CGG)_{9}$	31	193	Normal/Normal
55	$(CGG)_{24}$	24	24	$(CGG)_{9}AGG(CGG)_{15}$	25	51	Low/Low
56	$(CGG)_{9}AGG(CGG)_{13}$	23	49	$(CGG)_{10}AGG(CGG)_{20}$	31	60	Low/Normal
57	$(CGG)_{10}AGG(CGG)_{9}AGG(CGG)_{9}$	30	205	$(CGG)_{10}AGG(CGG)_{9}AGG(CGG)_{9}$	30	205	Normal/Normal
58	$(CGG)_{10}AGG(CGG)_{9}AGG(CGG)_{9}$	30	205	$(CGG)_{9}AGG(CGG)_{12}AGG(CGG)_{9}$	32	201	Normal/Normal
59	$(CGG)_{29}$	29	29	$(CGG)_{10}AGG(CGG)_{20}$	31	60	Normal/Normal
60	$(CGG)_{10}AGG(CGG)_{9}$	20	49	$(CGG)_{13}AGG(CGG)_{9}$	23	61	Low/Low
61	$(CGG)_{9}AGG(CGG)_{10}AGG(CGG)_{9}$	30	193	$(CGG)_{10}AGG(CGG)_{9}AGG(CGG)_{11}$	32	207	Normal/Normal
62	$(CGG)_{10}AGG(CGG)_{9}AGG(CGG)_{9}$	30	205	$(CGG)_{10}AGG(CGG)_{9}AGG(CGG)_{11}$	32	207	Normal/Normal
63	$(CGG)_{13}AGG(CGG)_{9}$	23	61	(CGG)9AGG(CGG)33	43	69	Low/High
64	$(CGG)_{10}AGG(CGG)_{9}AGG(CGG)_{9}$	30	205	$(CGG)_{10}AGG(CGG)_{9}AGG(CGG)_{9}$	30	205	Normal/Normal
65	$(CGG)_{9}AGG(CGG)_{9}AGG(CGG)_{9}$	29	189	$(CGG)_{10}AGG(CGG)_{9}AGG(CGG)_{9}$	30	205	Normal/Normal
66	$(CGG)_{10}AGG(CGG)_{9}AGG(CGG)_{9}$	30	205	$(CGG)_{9}AGG(CGG)_{12}AGG(CGG)_{9}$	32	201	Normal/Normal
67	$(CGG)_{10}AGG(CGG)_{9}AGG(CGG)_{9}$	29	205	$(CGG)_{10}AGG(CGG)_{9}AGG(CGG)_{9}$	29	205	Normal/Normal
68	$(CGG)_{10}AGG(CGG)_{9}$	20	49	(CGG)23	23	23	Low/Low
69	$(CGG)_{10}AGG(CGG)_{9}AGG(CGG)_{9}$	30	205	$(CGG)_{10}AGG(CGG)_{9}AGG(CGG)_{9}$	30	205	Normal/Normal
70	$(CGG)_{10}AGG(CGG)_{9}AGG(CGG)_{9}$	30	205	$(CGG)_{9}AGG(CGG)_{26}$	36	62	Low/Normal
71	$(CGG)_{9}AGG(CGG)_{19}$	29	55	$(CGG)_{10}AGG(CGG)_{9}AGG(CGG)_{9}AGG(CGG)_{9}$	39	829§	Normal/High

72	$(CGG)_9AGG(CGG)_{10}$	20	46	$(CGG)_{10}AGG(CGG)_9AGG(CGG)_{22}$	43	218	Normal/High
73	$(CGG)_{10}AGG(CGG)_9AGG(CGG)_9$	30	205	$(CGG)_{10}AGG(CGG)_{20}$	31	60	Low/High
74	$(CGG)_9AGG(CGG)_{13}$	23	49	$(CGG)_9AGG(CGG)_9AGG(CGG)_{22}$	42	202	Normal/Normal
75	$(CGG)_{12}AGG(CGG)_{10}$	23	58	$(CGG)_9AGG(CGG)_9AGG(CGG)_{32}$	51	212	Low/High
76	$(CGG)_9AGG(CGG)_{20}$	30	56	$(CGG)_9AGG(CGG)_{12}AGG(CGG)_9$	32	201	Low/High
77	$(CGG)_{10}AGG(CGG)_9AGG(CGG)_9$	30	205	$(CGG)_9AGG(CGG)_{30}$	40	66	Normal/Normal
78	$(CGG)_{13}AGG(CGG)_9$	23	61	$(CGG)_{10}AGG(CGG)_9AGG(CGG)_9$	30	205	Normal/High
79	$(CGG)_8AGG(CGG)_9$	18	41	$(CGG)_{10}AGG(CGG)_9AGG(CGG)_9$	30	205	Low/Normal
80	$(CGG)_{10}AGG(CGG)_{19}$	30	59	$(CGG)_{10}AGG(CGG)_9AGG(CGG)_9$	30	205	Low/Normal
81	$(CGG)_{10}AGG(CGG)_9$	20	49	$(CGG)_{10}AGG(CGG)_9AGG(CGG)_9$	30	205	Normal/Normal
82	$(CGG)_{25}$	25	25	$(CGG)_9AGG(CGG)_9AGG(CGG)_9$	29	189	Low/Normal
83	$(CGG)_{10}AGG(CGG)_9$	20	49	$(CGG)_{10}AGG(CGG)_9AGG(CGG)_{10}$	31	206	Low/Normal
84	$(CGG)_{10}AGG(CGG)_9$	20	49	$(CGG)_{10}AGG(CGG)_9AGG(CGG)_{11}$	32	207	Low/Normal
85	$(CGG)_{10}AGG(CGG)_9$	20	49	$(CGG)_9AGG(CGG)_9AGG(CGG)_9$	29	189	Low/Normal
86	$(CGG)_{10}AGG(CGG)_9$	20	49	$(CGG)_{10}AGG(CGG)_9AGG(CGG)_{10}$	31	206	Low/Normal
87	$(CGG)_{16}AGG(CGG)_9$	26	73	$(CGG)_{10}AGG(CGG)_9AGG(CGG)_{10}$	31	206	Low/Normal
88	$(CGG)_{23}$	23	23	$(CGG)_9AGG(CGG)_9AGG(CGG)_9$	29	189	Normal/Normal
89	$(CGG)_{25}$	25	25	$(CGG)_9AGG(CGG)_9AGG(CGG)_9AGG(CGG)_9$	39	765§	Low/Normal
90	$(CGG)_{10}AGG(CGG)_{19}$	30	59	$(CGG)_{10}AGG(CGG)_9AGG(CGG)_9$	30	205	Low/High
91	$(CGG)_{11}AGG(CGG)_9$	21	53	$(CGG)_{10}AGG(CGG)_9AGG(CGG)_{10}$	31	206	Normal/Normal
92	$(CGG)_{12}AGG(CGG)_{10}$	23	58	$(CGG)_9AGG(CGG)_{12}AGG(CGG)_9$	32	201	Low/Normal
93	$(CGG)_{10}AGG(CGG)_9$	20	49	$(CGG)_{10}AGG(CGG)_9AGG(CGG)_{10}$	31	206	Low/Normal
94	$(CGG)_{10}AGG(CGG)_9$	20	49	$(CGG)_{10}AGG(CGG)_9AGG(CGG)_9$	30	205	Low/Normal
95	$(CGG)_9AGG(CGG)_{10}$	20	46	$(CGG)_{10}AGG(CGG)_9AGG(CGG)_9$	30	205	Low/Normal
96	$(CGG)_{10}AGG(CGG)_9$	20	49	$(CGG)_{10}AGG(CGG)_9AGG(CGG)_9$	30	205	Low/Normal

97	$(CGG)_{10}AGG(CGG)_6$	17	46	$(CGG)_{10}AGG(CGG)_6AGG(CGG)AGG(CGG)_{10}$	30	750[§]	Low/Normal
98	$(CGG)_{10}AGG(CGG)_{15}$	26	55	$(CGG)_{10}AGG(CGG)_9AGG(CGG)_9$	29	205	Low/Normal
99	$(CGG)_{10}AGG(CGG)_9AGG(CGG)_9$	30	205	$(CGG)_9AGG(CGG)_{30}$	40	66	Normal/Normal
100	$(CGG)_9AGG(CGG)_{17}$	27	49	$(CGG)_9AGG(CGG)_{10}AGG(CGG)_9$	29	193	Normal/High
101	$(CGG)_{24}$	24	24	$(CGG)_{10}AGG(CGG)_9AGG(CGG)_9$	30	205	Normal/Normal
102	$(CGG)_9AGG(CGG)_9$	19	45	$(CGG)_{10}AGG(CGG)_9AGG(CGG)_9$	30	205	Low/Normal
103	$(CGG)_9AGG(CGG)_{12}$	22	48	$(CGG)_{10}AGG(CGG)_9AGG(CGG)_9$	30	205	Low/Normal
104	$(CGG)_{10}AGG(CGG)_9$	20	49	$(CGG)_{10}AGG(CGG)_9AGG(CGG)_9$	30	205	Low/Normal
105	$(CGG)_9AGG(CGG)_{10}$	20	46	$(CGG)_{10}AGG(CGG)_8AGG(CGG)_9$	29	201	Low/Normal
106	$(CGG)_{10}AGG(CGG)_9$	20	49	$(CGG)_9AGG(CGG)_9AGG(CGG)_9$	29	189	Low/Normal
107	$(CGG)_{10}AGG(CGG)_9$	20	49	$(CGG)_{10}AGG(CGG)_9AGG(CGG)_9$	30	205	Low/Normal
108	$(CGG)_{10}AGG(CGG)_9$	20	49	$(CGG)_{10}AGG(CGG)_9AGG(CGG)_9$	30	205	Low/Normal
109	$(CGG)_{10}AGG(CGG)_9$	20	49	$(CGG)_{10}AGG(CGG)_9AGG(CGG)_9$	30	205	Low/Normal
110	$(CGG)_9AGG(CGG)_{19}$	29	55	$(CGG)_9AGG(CGG)_9AGG(CGG)_9$	30	189	Normal/Normal
111	$(CGG)_{10}AGG(CGG)_9$	20	49	$(CGG)_{10}AGG(CGG)_9AGG(CGG)_{10}$	31	206	Low/Normal
112	$(CGG)_{10}AGG(CGG)_9AGG(CGG)_9$	30	205	$(CGG)_{20}AGG(CGG)_9$	30	89	Normal/Normal
113	$(CGG)_{10}AGG(CGG)_9AGG(CGG)_9$	30	205	$(CGG)_{10}AGG(CGG)_{42}$	52	82	Normal/High
114	$(CGG)_{23}$	23	23	$(CGG)_9AGG(CGG)_{12}AGG(CGG)_9$	32	201	Low/Normal
115	$(CGG)_{13}AGG(CGG)_9$	23	61	$(CGG)_9AGG(CGG)_9AGG(CGG)_9$	29	189	Low/Normal
116	$(CGG)_9AGG(CGG)_{14}$	24	50	$(CGG)_{10}AGG(CGG)_9AGG(CGG)_9$	30	205	Low/Normal
117	$(CGG)_9AGG(CGG)_{10}AGG(CGG)_{10}$	31	194	$(CGG)_{10}AGG(CGG)_{37}$	48	77	Normal/High
118	(CGG)20AGG(CGG)9	30	89	$(CGG)_{10}AGG(CGG)_9AGG(CGG)_9$	30	205	Normal/Normal
119	$(CGG)_{10}AGG(CGG)_{13}$	24	53	$(CGG)_{10}AGG(CGG)_9AGG(CGG)_{10}$	31	206	Low/Normal
120	$(CGG)_{13}AGG(CGG)_9$	23	61	$(CGG)_{10}AGG(CGG)_9AGG(CGG)_9$	30	205	Low/Normal
121	$(CGG)_{12}AGG(CGG)_{10}$	23	58	(CGG)9AGG$(CGG)_9AGG(CGG)_{11}$	31	191	Low/Normal

AGG, adenine-guanine-guanine; CGG, cytosine-guanine-guanine; *FMR1*, fragile X messenger ribonucleoprotein 1; x, number of CGGs; [§]Samples with three AGG interruptions; *Equivalent* (white background) and *dissimilar* (gray background) groups.

Supplementary Table S3. Correlation between the *allelic score* of allele 2, markers of ovarian reserve, and IVF outcomes for *equivalent* and *dissimilar* groups.

	***Equivalent* group**			***Dissimilar* group**		
	**Pearson´s correlation coefficient**	***p*-value**	***n***	**Pearson´s correlation coefficient**	***p*-value**	***n***
**Markers of ovarian reserve**						
Day 3 FSH (mUI/ml)	-0.0280	0.825	65	0.00533	0.970	51
AMH (ng/ml)	-0.119	0.349	64	-0.0402	0.791	46
AFC	0.122	0.455	40	-0.0934	0.605	33
**IVF outcomes**						
**Response to ovarian stimulation**						
Total dose of gonadotrophins (IU/ml)	0.0417	0.736	68	-0.0822	0.567	51
Stimulation duration (days)	0.0199	0.872	68	0.0434	0.760	52
Number of follicles on the trigger day	0.0399	0.747	68	-0.0162	0.910	51
Number of retrieved oocytes	-0.101	0.414	68	0.0231	0.872	51
Number of immature oocytes	0.0522	0.672	67	0.152	0.299	49
Number of aberrant oocytes	0.0315	0.799	68	0.210	0.148	49
**Oocyte maturation**						
Number of injected MII oocytes	-0.0913	0.459	68	0.000245	0.999	49
**Fertilization success**						
Number of 2PN oocytes	-0.149	0.240	64	-0.0959	0.517	48

2PN, two pronuclei; AFC, antral follicle count; AMH, anti-Müllerian hormone; FSH, follicle-stimulating hormone; MII, metaphase II; n, number of samples.

Supplementary Table S4. Detailed description of markers of ovarian reserve and IVF outcomes for each sample.

Sample	Day 3 FSH (mIU/ml)	AMH (ng/ml)	AFC	Total dose of gonadotrophins (IU/ml)	Stimulation duration (days)	Number of follicles on the trigger day	Number of retrieved oocytes	Number of immature oocytes	Number of aberrant oocytes	Number of injected MII oocytes	Number of oocytes with 2PN
1	5.3	4.2	4	2400	11	7	33	5	4	24	15
2	6.2	1.5	6	3300	11	11	27	7	2	18	8
3	5.7	4.4		1025	7	11	22	2	0	10	8
4	6.1	2.1	5	4500	11	11	14	3	0	11	8
5	4.2	2.7		2400	11	7	9	0	0	9	8
6	10.6	0.7		5550	13	12	13	1	1	12	8
7	7.5	1.9	12	2175	10	11	22	5	0	17	7
8	12.9	0.6	4	4800	10	1	2	0	0	2	1
9	4.9	2.8	7	1575	7	5	15	8	0	7	6
10	6.8	1.4	5	3600	12	2	3	0	0	3	2
11	5.1	1.4	12	2700	9	3	3	1	0	2	1
12	6.3		5	4725	11	3	1	0	1	0	NA
13	4	4.9		2925	13	16	21	3	12	6	3
14		0.9		4500	15	4	2	0	1	1	1
15	10.7	0.8	4	3300	11	5	3	2	0	1	0
16	8	1.4	5	3450	12	6	3	1	0	2	2
17	6.57	11.2		2475	11	7	4	1	0	3	2
18	8.6	1.5	12								
19	5			2450	10	13	5	0	0	5	0
20	7		8	2900	9	11	24	6	2	16	5
21	5.2	4.5		1650	11	5	10	1	0	9	5
22	13.9	0.1	1	3150	11	1	1	0	0	1	1
23	6	2.6		1800	8	8	10	3	2	5	3
24	9.8	3.4	8	3500	12	21	26	4	1	21	17
25	7.5	4.7		2625	12	8	11	2	0	9	7

26		1.6	3	2700	12	7	10	3	0	7	6
27	5.4	2.6		1800	8	5	14	4	1	9	5
28	5.2	2.4	12	3150	14	11	17	0	0	17	14
29	9.4	0.7	6	3000	10	5	7	1	1	5	5
30	7.4	1.3	8	3750	13	3	5	0	1	4	4
31	11	0.6	8	2100	7	1	1	0	0	1	1
32	4.1	3.9		1575	7	12	25	7	4	14	5
33	7.5	0.7	4	7200	16	2	3	0	0	3	2
34	7.3	4.9		2250	10	6	8	0	0	8	5
35	9.6	3.0		3000	10	5	11	2	1	8	7
36	5.7	3.1	8	3000	10	11	8	2	0	6	4
37	7.2	3.1	16	2250	10	3	10	1	2	7	4
38	7.1	1.3		2250	10	5	12	1	0	11	6
39	3.3	5.2		2700	9	8	17	0	0	17	8
40	5	4.8		2025	9	5	13	5	6	8	2
41	5.9	6.2	5	2475	11	16	24	4	2	19	16
42	11.6	0.7	6	3000	10	6	7	1	1	5	2
43	10.3	2.2	6	3600	12	4	2	2	0	0	NA
44	7.4		8	1650	6	4	4	0	1	4	2
45	9.1	0.9	5	4200	14	5	10	1	0	9	5
46	5.1	5.5		1525	12	12	16	3	2	11	7
47	5.5	8.2		2250	10	6	14	2	2	10	8
48		2.8	4	2400	8	0	13	6	0	7	5
49	8.8	2.7	13	2250	10	0	7	1	1	6	3
50	7.3	3.5		3162.5	12	12	22	3	1	18	11
51	4.8	1.0	8	3600	12	5	9	1	0	8	5
52	4.9	1.5	8	2400	8	4	7	0	1	6	3
53	6.7	2.6		2700	12	10	14	1	1	12	8
54	9	2.9	8	2887.5	11	3	5	2	0	3	1
55	7.7	0.8	4	3300	11	3	2	0	0	2	2

56		0.3	2	1800	6	1	1	0	1	0	NA
57	7.6	2.3	10	2850	10	4	1	0	0	1	0
58	6.7	1.1	7	3300	11	5	4	1	0	3	2
59	10.5	1.0	6	2100	7	0	1	1	0	0	NA
60	8.5	0.5	3	3000	10	1	3	0	1	2	1
61	6.1			1750	10	19	46	4	0	28	16
62	11.6	1.7		2250	10	6	10	3	0	7	5
63	4.4	10.9		2025	9	13	25	3	2	10	8
64	8	1.9		3300	12	10	9	2	0	7	6
65	7.4	3.7		2700	12	12	16	1	2	13	9
66	7.3	6.7		2500	10	11	6	0	0	6	5
67	7	7.0		1350	9	11	23	1	0	11	7
68	6.4	5.6		1650	11	0	16	1	1	6	6
69	11.7	1.4	10	3000	10	8	10	1	1	8	6
70	8.1	2.7	10	1750	12	8	11	1	1	9	5
71	12.5	0.8		2475	11	5	8	3	0	5	3
72	8.4	1.5	6	2100	9	2	9	2	0	7	3
73	7			1800	6	3	4	3	0	1	1
74	4.7	10.5		2025	9						
75	5.3	2.3	14	3300	12	8	12	0	0	12	9
76	6.6		5	1800	10	14	18	1	0	17	11
77	6.7			1300	8	6	6	0	0	6	3
78	7.4	3.3	14		8	8	21	2	0	4	0
79	8.8	1.5	4	2400	8	3	3	0	0	3	1
80	9.3	1.1	10	2250	10	2	6	1	0	0	NA
81	10.9	2.8	14	1800	8	4	14	6	1	7	3
82	10.9	0.6	4	3300	11	5	5	1	0	4	4
83	5.5	2.6	8	3300	12	2	5	0	0	5	4
84		4.3	10	2025	9	5	10	2	1	8	6
85	5	2.8		2700	12	6	6	0	0	6	5

86	6.7	1.7	10	1950	7	4	6	0	0	6	4
87	8.4	5.3		1875	9	11	15	2	0	13	5
88	5.9	3.3		1800	8	10	20	4	1	15	11
89	4.1	11.8		1800	12	14	20	4	2	14	9
90	9	2.7		2025	9	12	11	1	0	10	1
91	7.5	0.9	4	2700	9	6	7	3	0	4	3
92	9.4	3.7	12	3000	10	8	15	4	1	10	5
93	6.4	1.6		1975	10	12	24	7	0	17	13
94	6.2	0.9	6	3900	13	0	7	0	0	7	2
95	6.7	4.0	14	2700	9	10	13	6	2	5	4
96	11.6	2.0	7	3300	11	6	13	3	8	10	1
97	6.3	2.7	5	1500	10	10	18	4	1	14	10
98	8.4	4.8		2925	13	8	14	6	6	2	2
99	8.1	2.4	9	3000	11	8	16	4	1	11	9
100	7.5	4.8		3050	14	16	6	1	2	3	2
101	6.1			2000	10	18	28	2	1	6	5
102	4.9	10.2		1700	12	21	25	0	0	13	12
103	4.7	6.0		3175	12	7	24	0	0	24	17
104	4.6	1.7		3300	11	11	15	3	1	11	7
105	6.24	2.5	10	2200	10	7	9	4	1	4	3
106	4.5	7.2	5	1500	5	2	0	NA	NA	NA	NA
107	9.1	5.9	9	2200	9	11	25	9	2	14	9
108	133	2.3	8	2500	10	6	5	1	0	4	1
109	12.2	0.8	2	3000	10	1	0	NA	NA	NA	NA
110	9.5	1.7	6	3600	12	5	6	0	0	6	2
111	7.4	18.9		2250	10	14	32	1	0	13	9
112	7.7	1.1	7	3000	10	6	8	1	0	7	3
113	8.3	1.8	7	3000	10	9	10	0	1	4	4
114	8.1	4.7		2350	12	11	16	3	0	13	12
115	6.2	2.2	5	3000	10	3	6	0	0	6	1

116	6.7	2.9	7	2700	9	0	11	4	0	7	7
117	6	1.3	6	2100	7	4	5	1	0	4	3
118	10.2		4	2400	8	2	6	0	0	6	3
119	5.24			2475	11	18	15	0	0	8	2
120	4.9	1.2	6	3000	10	2	1	0	0	1	1
121	6.1	4.6	12	1800	8	14	20	1	2	17	7

2PN, two pronuclei; AFC, antral follicle count; AMH, anti-Müllerian hormone; FSH, follicle-stimulating hormone; LH, luteinizing hormone; MII, metaphase II; NA, not applicable.
Equivalent (white background) and *dissimilar* (gray background) groups.

Supplementary Table S5. Fluorescent PCR components and respective final concentration.

**Reagents**	**Final concentration**
AccuTaq™ LA 10x Buffer (Sigma-Aldrich®, St. Louis, Missouri, USA)	1 x
Betaine (Sigma-Aldrich®)	1 M
dATP/dCTG/dTTP (Bioline, London, UK)	0.12 mM
dGTP (Bioline)	0.024 mM
7-Deaza-dGTP (Roche®, Basel, Switzerland)	0.44 mM
DMSO (Sigma-Aldrich®)	4.80%
P1- g. *FMR1*_CGG_F (Thermo Fisher Scientific, Waltham, Massachusetts, USA)	0.6 pmol/µl
P2- g. *FMR1*_CGG_R* (Thermo Fisher Scientific)	0.6 pmol/µl
AccuTaq™ LA DNA Polymerase (Sigma-Aldrich®)	0.08 U/µl
gDNA	150 ng
dH_2O	Up to 25 µl

CGG, cytosine-guanine-guanine; dATP, deoxyadenosine triphosphate; dCTG deoxycytidine triphosphate, dGTP, deoxyguanosine triphosphate; dH_2O; distilled water; DMSO, dimethyl sulfoxide; dTTP, 2′-deoxythymidine 5′-triphosphate; F, forward; *FMR1*, fragile X messenger ribonucleoprotein 1; gDNA, genomic DNA; LA, long and accurate; P1; primer 1; P2, primer 2; R, reverse; UK, United Kingdom; USA, United Stated of America.

Supplementary Table S6. Triplet-primed-PCR (TP-PCR) components and respective final concentration.

**Reagents**	**Final concentration**
PCR Master Mix (Promega®, Madison, Wisconsin, USA)	1 x
Betaine (Sigma-Aldrich®, St. Louis, Missouri, USA)	0.6 M
7-Deaza-dGTP (Roche®, Basel, Switzerland)	0.4 mM
Q-Solution® (Qiagen®, Hilden, Germany)	0.5 x
DMSO (Sigma-Aldrich®)	10%
P1- g. *FMR1*_TP-PCR_F (Thermo Fisher Scientific, Waltham, Massachusetts, USA)	0.4 pmol/µl
P2- g. *FMR1*_TP-PCR_GCC* (Thermo Fisher Scientific)	0.2 pmol/µl
P3- g. *FMR1*_TP-PCR_R (Thermo Fisher Scientific)	0.2 pmol/µl
gDNA	150 ng
dH_2O	Up to 25 µl

dGTP, deoxyguanosine triphosphate; dH_2O; distilled water; DMSO, dimethyl sulfoxide; F, forward; *FMR1*, fragile X messenger ribonucleoprotein 1; gDNA, genomic DNA; P1, primer 1; P2, primer 2; PCR, polymerase chain reaction; R, reverse; TP, triplet-primed; USA, United Stated of America.

Supplementary Figures

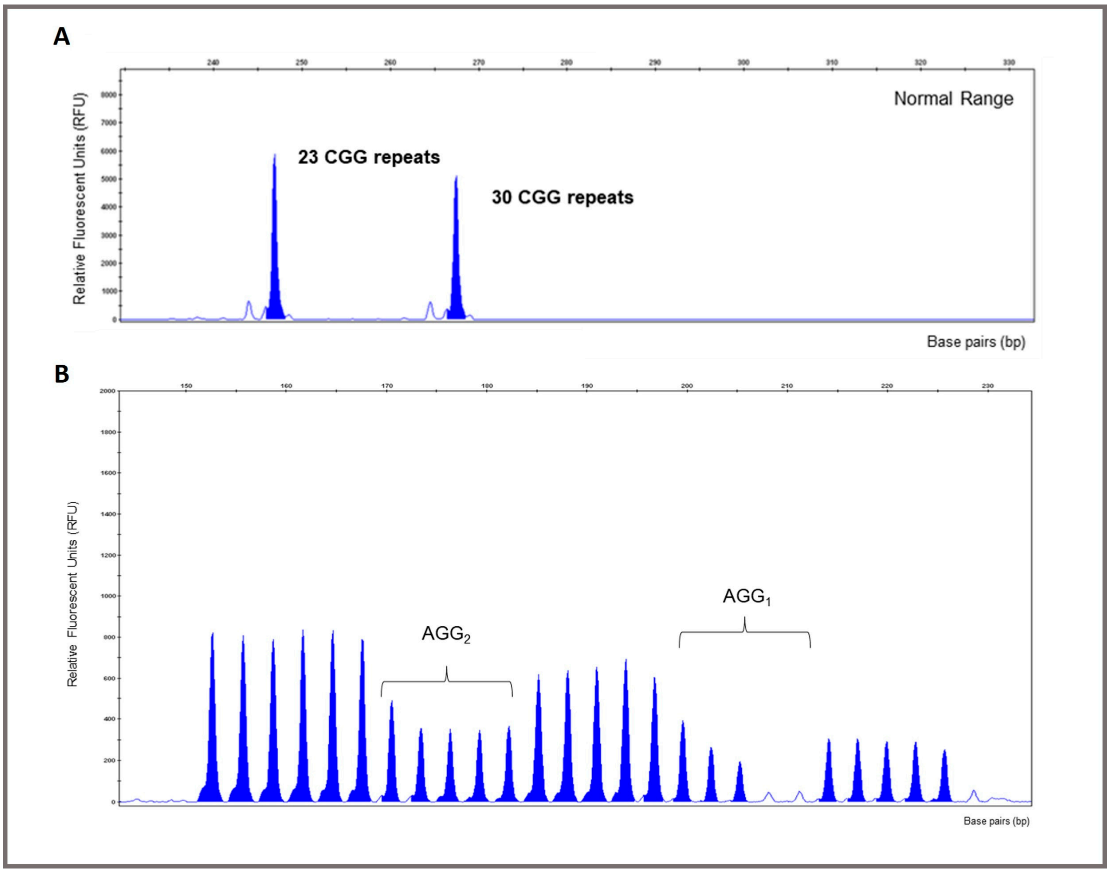

Supplementary Figure S1. Representative electropherograms of fluorescent PCR (A), and FAM-labelled TP-PCR (B) for a heterozygous sample carrying 23 and 30 CGG repeats (A). FAM-labelled TP-PCR (B) indicated two AGG interruptions, consistent with a $[CGG]_{10}AGG[CGG]_9AGG[CGG]_9$ pattern for the allele with 30 repeats, and $[CGG]_{23}$ uninterrupted pattern for the allele with 23 repeats.
